# Pupil Dilation Co-Varies with Memory Strength of Individual Traces in a Delayed Response Paired-Associate Task

**DOI:** 10.1371/journal.pone.0051134

**Published:** 2012-12-05

**Authors:** Hedderik van Rijn, Jelle R. Dalenberg, Jelmer P. Borst, Simone A. Sprenger

**Affiliations:** 1 Department of Psychology, University of Groningen, Groningen, The Netherlands; 2 Department of Artificial Intelligence, University of Groningen, Groningen, The Netherlands; 3 Department of English Language and Linguistics, University of Groningen, Groningen, The Netherlands; University of California, Davis, United States of America

## Abstract

Studies on cognitive effort have shown that pupil dilation is a reliable indicator of memory load. However, it is conceivable that there are other sources of effort involved in memory that also affect pupil dilation. One of these is the ease with which an item can be retrieved from memory. Here, we present the results of an experiment in which we studied the way in which pupil dilation acts as an online marker for memory processing during the retrieval of paired associates while reducing confounds associated with motor responses. Paired associates were categorized into sets containing either 4 or 7 items. After learning the paired associates once, pupil dilation was measured during the presentation of the retrieval cue during four repetitions of each set. Memory strength was operationalized as the number of repetitions (frequency) and set-size, since having more items per set results in a lower average recency. Dilation decreased with increased memory strength, supporting the hypothesis that the amplitude of the evoked pupillary response correlates positively with retrieval effort. Thus, while many studies have shown that “memory load” influences pupil dilation, our results indicate that the task-evoked pupillary response is also sensitive to the experimentally manipulated memory strength of individual items. As these effects were observed well before the response had been given, this study also suggests that pupil dilation can be used to assess an item’s memory strength without requiring an overt response.

## Introduction

The size of the pupil has repeatedly been shown to vary systematically with cognitive effort (see e.g., [1] for a review). One component of cognitive effort that has been associated with pupil dilation from the onset of research into the pupillary response is memory load (e.g., [2]). Over the years, this work has established a clear link between memory encoding and pupil dilation. For example, Van Gerven et al. [3] and Karatekin [4] let participants memorize lists of serially presented digits and observed an increase in pupil dilation for each additional digit, and Granholm [5] showed that the pupil dilated with increased sequence length in digit span tasks until all cognitive capacity is used, after which the pupillary response decreased.

Another potential source of cognitive effort as indexed by the pupillary response is retrieval effort (or retrieval attempt [6], a term that “refers to the mobilization of processing resources in service of a retrieval attempt and [that] is operationalized in terms of relative difficulty, the assumption being that the more difficult the retrieval task, the greater the effort expended” [7], p. 583, see also [8]). This relative difficulty is typically deduced from reaction times and accuracy measures that are thought to reflect the strength of the memory traces on retrieval processes (e.g., [9]) and the influence of contextual effects (e.g., [10], [11]). However, this link is not necessarily bidirectional, since studies [12], [13] have shown that conditions controlled for reaction time can show differential pupillary responses. Nevertheless, both in computational models (see [14] for a review) and in experimental designs (e.g., [15]) the strength of memory traces is often operationalized by manipulating the frequency (the number of repetitions within an experiment) and age or recency (the time since the last presentation of an item). Thus, items that are less often rehearsed or that were presented longer ago are thought to have a weaker memory strength, making them more difficult to retrieve than items with a higher frequency or those that were presented more recently. In the present study, we varied frequency and recency to test whether retrieval-evoked pupil dilation is sensitive to the relative strength of the retrieved item’s memory trace.

Earlier work has shown that pupil dilation indeed fluctuates with memory strength. For example, Hyöna, Tommolaa and Alajaa [16] asked for the translation of either difficult or easy aurally presented words and observed increased pupil dilations for the more difficult words. Similarly, in a visual task, Kuchinke, Võ, Hofmann and Jacobs [17] have shown that low frequent words evoke a stronger pupillary response than high frequent words during a lexical decision task. Although it has been argued that a lexical decision can be made before the presented letter string has completely been retrieved from memory (e.g., [18], [19]), the observed effect of the manipulation of frequency argues in favor a frequency-based component in the pupillary response evoked by a memory retrieval.

In these studies, the pupillary response was measured between the presentation of the stimulus and the response, combining potential effects of response selection driven by the memory retrieval and response execution. As response execution is known to influence pupillary responses (e.g., [20]–[23], with motor preparation accounting for up to 70% of the observed pupil dilation in certain studies [24]), a veridical assessment of the influence of memory strength on pupillary responses can only be achieved without a motor response (or with a delayed response). This is especially relevant since motor preparation is affected by the current state of the decision process [25] and by response competition [26], aspects that might differ for low and high frequent items.

Given that the above-mentioned studies provide indirect evidence for the view that both frequency and recency of prior encounters affect the evoked pupillary response, we designed an experiment to test the hypothesis that the pupillary response is a reliable indicator of retrieval effort while controlling for the possible confounds associated with the motor response. To experimentally manipulate frequency and recency during the experiment, we presented participants with sets of paired associates in blocks of either four or seven pairs. After the initial presentation of a cue-answer pair, rehearsals consisted of a slow-paced presentation of the retrieval cue, after which participants had to provide the associated answer by moving the mouse to a location associated with the retrieval cue. The number of repetitions of an item determined the current frequency, and the number of items in a set determined the average recency (more items in a set result in a wider spacing between repetitions and thus a lower average recency). By presenting the retrieval cue for six seconds before a response could be given, we could measure the pupillary response evoked by the cue-induced retrieval processes while reducing potential contamination caused by the physical response.

We hypothesized that the retrieval cue-evoked pupillary response would decrease with each repetition, since every repetition would strengthen the associated memory representation. Moreover, the pupillary response to items from a small set was expected to be smaller than the response to items from a large set, since the longer average delay between repetitions should result in reduced memory strength for the items from a large set.

## Methods

### Participants

Nineteen first year Psychology students of the University of Groningen participated in exchange for study credits. Data of 4 participants were not analyzed because of excessive eye blinks or missing dilation data during the critical parts of the trials (i.e., the presentation of the retrieval cue), leaving data of 15 participants (5 male; average age 21.5 years; range 18–24).

### Ethics Statement

Informed consent as approved by the Ethical Committee Psychology (#10072-E) of the University of Groningen was obtained before testing.

### Stimuli & Design

**Figure 1:**
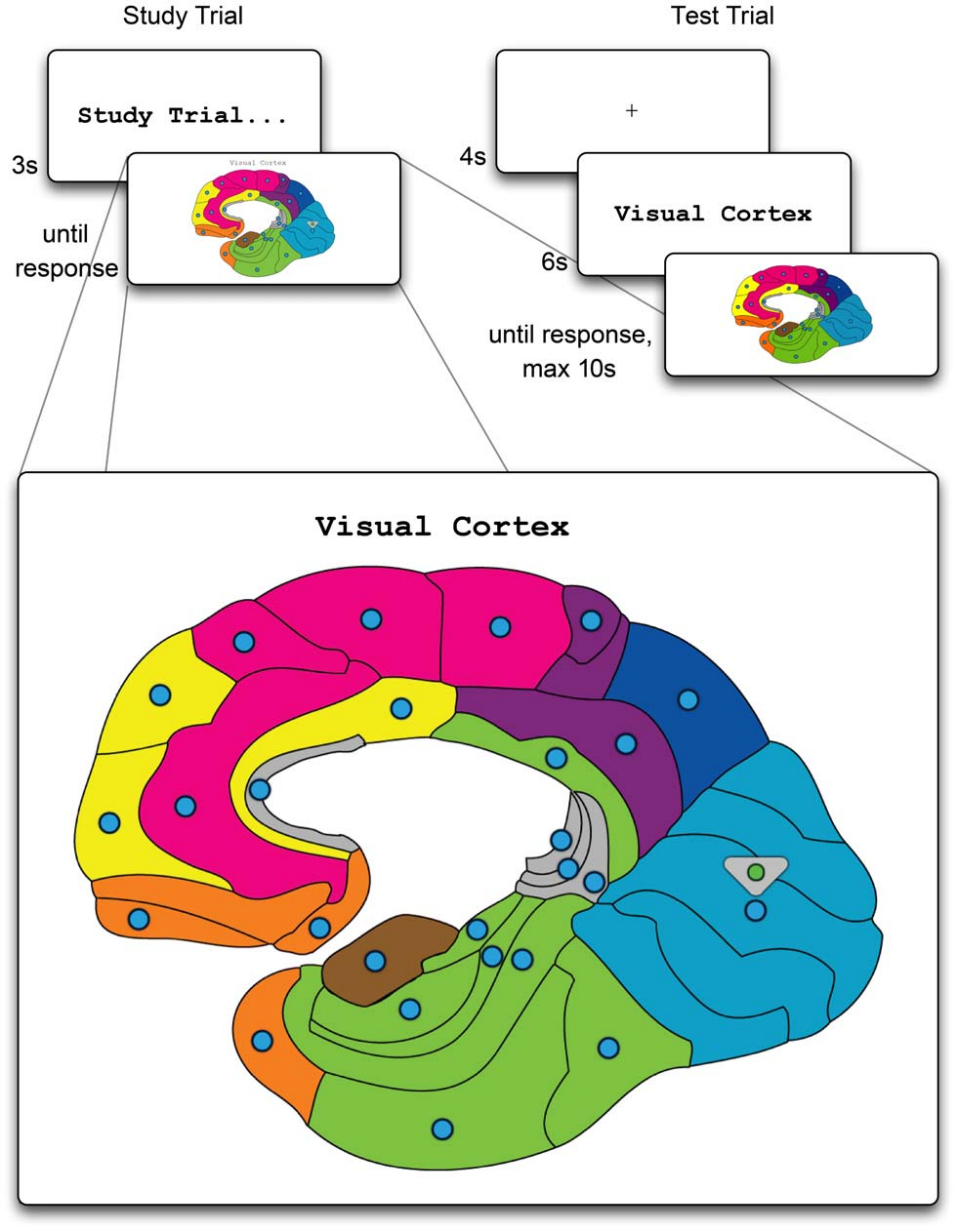
Example of a study trial (left side) in which a new paired-associate is presented, and a test trial (right side) during which the pupil dilation is measured. Note that after the test trial a feedback screen (not shown) was provided. The zoomed-in bottom part of the figure shows the learning screen with the name of the area being presented on the top of the screen, and a small triangle indicating the corresponding circle/area.

The experiment was set up as a brain-topography learning session. Stimuli were 26 paired associates consisting of the topographical full name of a brain area (e.g., “Inferior Temporal Gyrus”) and the location of that brain area indicated by a circle on a cross-section of the brain (see Figure 1). The areas largely correspond to Brodmann areas. Although freshman psychology students are likely to be familiar with some of the areas from earlier courses, the materials had not yet been explicitly covered in the participating students’ curriculum. Accordingly, the participants did not report high levels of familiarity with the materials during debriefing.

Each participant saw all 26 items, randomly distributed across five sets, consisting of three sets of four and two sets of seven items. Together, the initial presentation of a given set of items (study trials) and their subsequent four repetitions (test trials) formed one experimental block, with the repetitions allowing for studying the effect of frequency on the pupillary response. The first startup block was always a short retention block and was not analyzed. The subsequent experimental blocks alternated between long and short blocks (i.e., (S), L, S, L, S). This experimental manipulation allows for studying the overall effect of retention interval by means of comparing the long and short blocks.

Within sets, the order in which the test trials were presented was randomized in such a way that a given item was never presented twice in a row. The blocks consisting of four items per set constituted the short retention interval condition, and the blocks consisting of seven items constituted the long retention interval condition: The average recency of the last encounter of any presented item was 4 in the short and 7 in the long condition. However, although the randomized presentation order removes any order-based predictability, it also results in a potential confound with respect to the operationalization of recency, which we will return to in the discussion.

### Apparatus and Setup

Participants were tested individually, and were seated in a dimly lit, small windowless room, containing a desk on which the monitor and the eye-tracker were located and to which a chinrest was attached, and a chair. The distance from the monitor (a 22″ IIlyama Vision Master Pro 513 CRT monitor set to a resolution of 1280×1024, 85 Hz) to the chinrest with forehead support (SR-Research Head Support), was 59 cm. The room was illuminated using two ceiling-attached lamps, resulting in ambient light levels of 5.5 lm/m^2^ as measured just below the forehead support (using a Testo 545 lux meter). This level of lighting was chosen to provide a comfortable level of lighting to the participants, while at the same time preventing mechanical muscle saturations at either extremely high or low levels of illumination (c.f., [27]). An additional source of light was the monitor, as the light grey background, on which all instructions and stimuli were presented, increased the light level measured at the forehead support to 14.0 lm/m^2^. Eye position and pupil dilation of the right eye was measured at 500 Hz using a dark pupil/corneal reflection SR Research EyeLink 1000 eye tracker (http://www.sr-research.com/EL_1000.html) placed immediately below the computer screen. Pupil dilation is measured in arbitrary units as recorded by the eye-tracker, which are linear in true diameter [28]. This eye tracker can measure pupil diameter with a resolution of 0.2% of diameter, corresponding to a resolution of 0.01 mm for a 5 mm pupil, and has a spatial resolution of <0.01° RMS.

Presentation of all stimuli was controlled with Matlab 2008 running on OS X 10.6, using Psychtoolbox (version 3.0.8) and Eyelink (version March 2009) extensions [29]. Before starting the actual experiment, a randomized target order 9-point (HV9) calibration routine was performed and a separate validation was performed using the EyeLink 1000 software. At the start of each block, a drift check was performed.

### Procedure

At the start of the experiment, participants were seated at the desk and read and signed the informed consent form. The chair and chinrest/forehead support were adjusted to the participant and the eye tracker was prepared for recording. Participants were told that they were to learn brain topography, and that they would get a set of study trials that presented the areas and the associated names, followed by four runs of test trials. All instructions were presented on the computer monitor.

A study trial, shown on the left of Figure 1, started with the string “Study trial…” being shown in the center of the screen for three seconds. The next screen contained the cross-section of the brain with an area name centered above the brain (shown at the bottom of Figure 1). An arrow indicated the associated area. Participants were instructed to memorize the name and associated area, and click the highlighted circle in the area to continue to the next trial.

**Figure 2:**
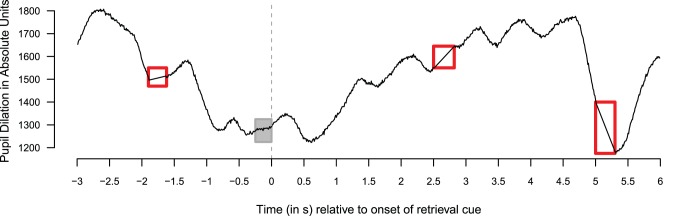
Typical raw pupillary response measured during a first test-trial. Red boxes indicate regions where the pupillary response was linearly interpolated; grey box indicates baseline used to calculate relative pupil dilation (see Figure 4).

The right side of Figure 2 shows a test trial. Each test trial started with a black fixation cross (a “+” in Courier New 26 point font, 6.5 mm wide) that was presented centered on a light-grey screen for 4 seconds. The light level measured during the presentation of the fixation cross was 14.0 lm/m^2^. After the fixation, the area name was presented, centered on the screen, in Courier New 26 point font in black on a light grey background. The length of the area name ranged from 13 (“Visual Cortex”) to 34 (“Ventral Posterior Cingulate Cortex”) mono-spaced characters, with 1.54 characters per cm. The light level measured during the presentation of the area name depended on the length in characters of the name, but was never lower than 13.5 lm/m^2^. The maximum contrast, defined as the ratio of amplitude of the stimulus to mean luminance as used by Chua [30], was (stimulus luminance - background luminance)/(stimulus luminance+background luminance) = (13.5–14.0)/(13.5+14.0) = -.018. After 6 seconds, the cross-section was shown again, but this time without the caption and arrow, and a response could be given by clicking on one of the 26 circles. If no response was given after 10 seconds, the experiment continued with this trial marked as incorrect. The light level measured at the response screen was 11.5 lm/m^2^, resulting in a contrast of (11.5–14.0)/(11.5+14.0) = −.098. After a response was given, feedback was provided (the feedback screens are not depicted in Figure 1). When the response was correct, the selected circle turned green for 1 second. When the response was incorrect, the selected circle turned red, and a green circle and an arrow indicated the correct area for 3 seconds. After feedback, the next trial started. The complete experiment, including setup and debriefing, lasted approximately 45 minutes.

### Measurement and Preprocessing of Pupillary Data

The slow pace of the experiment allowed for measuring the relatively slow fluctuations in pupil dilation. The presentation of the fixation cross for 4 seconds at the start of each test trial attenuated possible effects of the previous trial on pupil dilation and the six second presentation of the area name allowed for measuring a complete task-evoked pupil response while limiting the influence of any response preparation effects on dilation.

However, the long presentation of the area name combined with the length of the area names increases the probability of saccades (and potentially blinks) during which pupillary data is unreliable or which might have influenced pupillary measurements (e.g., [24], [31]). Saccades and blinks were detected online by the EyeLink software based on the gaze position, with a minimum velocity threshold set to 30°/sec, the motion threshold set to 0.1°, and the saccade acceleration threshold set to 8000°/sec^2^ (as recommended in Section 4.3.9. of [32]).

Prior to analysis, all pupillary data were preprocessed (for similar procedures, see e.g., [33], [34]). First, automatically detected saccade and eye blink induced artifacts were discarded and replaced by linear interpolation after extending the rejection area with 25 samples on both sides for saccades, and 50 samples on both sides for blinks to exclude pre- and post-saccade and blink artifacts. In addition to this automatic rejection procedure, all trials were visually inspected and all remaining artifacts were replaced by linear interpolation. Over all participants, 58 trials (3.9%) were completely excluded because of extensive blinks or tracking loss. This manual process was conducted blind with respect to experimental condition, response, and behavioral outcome (see p. 147 of [35]).

Figure 2 shows the raw pupillary data of the first experimental trial (the first test-trial presentation of the item “Ectosplenial Cortex”) of a single participant. The red vertical line indicates the presentation of the area name, which served as retrieval cue for the later response. The grey boxes indicate two regions in which pupillary information was missing for a longer period of time, and thus replaced by linear interpolation.

The saccades are also indicators of changes in gaze position, and therefore of gaze-position-dependent changes in the measured pupil size [36]. This effect will be larger for long area names than for short area names. Instead of reducing the effects of gaze position of pupil dilation by mathematical approximation [36], we included a random effect for items in the statistical analyses (see next section), accounting for pupillary effects that are specific to each item.

Next, pupil dilation was down-sampled from 500 Hz to 50 Hz. To allow for the comparison between participants and to correct for any tonic changes in pupil dilation over the scope of the experiment, absolute pupil dilation (as measured in arbitrary units) was converted into relative dilation expressed as a proportional difference from a baseline. The baseline was defined as the last 250 ms before the first presentation of a retrieval cue of a particular item. During this period, the fixation cross was displayed. Note that in cognitive research, reporting relative pupillary responses is common since absolute changes are often of less interest than the differential effects of the within-participant manipulated variables (e.g., [3], [4], [28], [37]–[41], and see [31] for a comparison between relative and absolute pupil dilation measurements and [42] for a review in which both absolute and relative measures are included).

Based on these relative dilation measures, the task-evoked pupil response is expressed as the difference between maximum constriction and maximum dilation per trial. These values were estimated by calculating the mean dilation in a window of 20 samples around the most extreme values within the first 3 seconds after onset of the retrieval cue. This range was chosen as visual inspection of the averaged data indicated that the maximum dilation was reached within the first three seconds. Moreover, inspection of the gaze positions indicated that during the first three seconds, the participants remained mainly focused on the presented retrieval cue. This retrieval-cue evoked pupil response will be used in all subsequent dilation-based analyses.

### Statistical Analysis

Since the items that we used in this experiment were selected from a finite set of possible items, it is important to account for the possible differences between items (due to familiarity, orthographic complexity, etc.) when accounting for changes in pupil dilation, reaction times, and accuracy (e.g., [43]). Moreover, since the items could not be controlled for length, the gaze patterns for fixations on the retrieval cues will differ per item, which in turn influences pupil dilation [36]. To account for these item-related effects, we analyzed all data using linear mixed effect models (also known as hierarchical models). By including both participant and item as random effect, effects associated with individual participants and items are accounted for. An extensive introduction to this method can be found in [43], [44].

In all analyses, we used binary coding for the retention set factor, with the long set coded as 0, and the short set coded as 1. The repetition factor is coded as integers, with the first repetition coded as 0. Based on this coding scheme, the intercept represents the estimated pupillary response evoked by items in the long retention set during their first repetition and all other estimates are expressed as differences to this intercept.

For the pupil dilation and reaction time analyses reported here, we used the maximum-likelihood-based linear mixed models provided via the lmer-function from package lme4 (version 0.999375-42) [45] in R (www.r-project.org, version 2.15.0). We will report p-values and upper and lower 95% highest posterior density (HPD95) intervals [44], obtained by Markov Chain Monte Carlo sampling (10,000 samples, using the package languageR, version 1.4, [46]). The HPD intervals can be interpreted as traditional 95% confidence intervals, demarcating the minimum and maximum value of the expected range of the underlying parameter. For each of the analyses, we started with the most complex model (i.e., containing all main effects and interactions), and constructed the best-fitting model based on log-likelihood-based stepwise model selection [47]. If a reduced model was preferred, we will report the associated log-likelihood statistics. For the binomial accuracy analyses, we used generalized linear mixed models using a logit-link function fit by a Laplace approximation of the likelihood and will report the estimated parameters and associated z-scores and p-values.

Since their introduction to the domain of psychophysiology (e.g., [47]) these analysis techniques have successfully been used to analyze ERPs (e.g., [48]), slow electrophysiological potentials (e.g., [49]), pupil dilation (e.g., [31]) and combinations of these measures (e.g., [50]).

## Results

Thirteen trials (<1%) were associated with a response time shorter than 500 ms or longer than 8 seconds, and were removed from all further analyses.

### Behavioral Data

**Figure 3:**
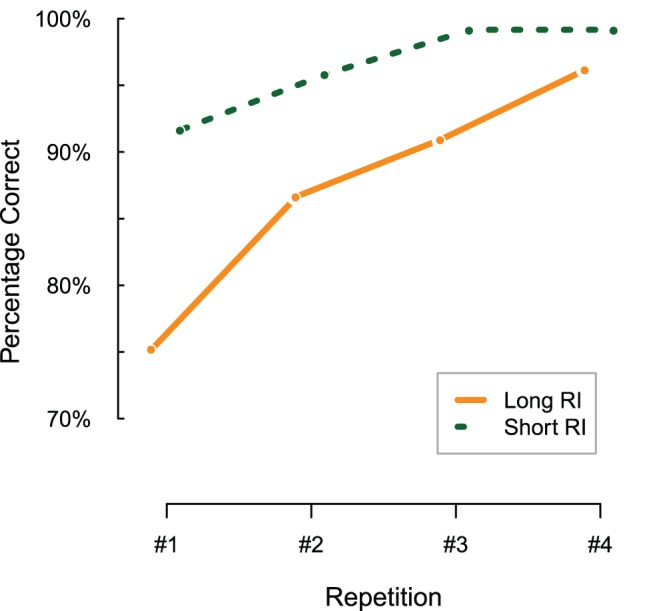
Percentage correct responses for the four repetitions, plotted separately for the long (orange, solid) and short (green, dashed) retention interval (RI) sets.

The main goal of the analysis of the behavioral data (i.e., accuracy and reaction times) is to assess whether our operationalization of memory strength was successful. Figure 3 shows the percentage correct responses for repetitions 1 to 4.

Correctness, measured binary per trial, was submitted as dependent variable to a binomial linear-mixed effect model, with the factors retention (long vs. short) and repetition (0 to 3) as fixed effects, and participant and item as random effects. Although inspection of Figure 3 might suggest an asymptote effect at later repetition lags, model comparisons indicated that including a term for the interaction between retention and repetition was not warranted (χ^2^(1) = 0.49, p = 0.483). As expected, the proportion of correct responses is higher in the short retention set condition (the difference between the long and short retention set is estimated at β = 1.50; z = 4.9; p<0.001, relative to an offset representing the long retention set of 1.82; z = 4.4; p<0.001) and increases with the number of repetitions (β = 0.83; z = 7.2; p<0.001).

**Figure 4:**
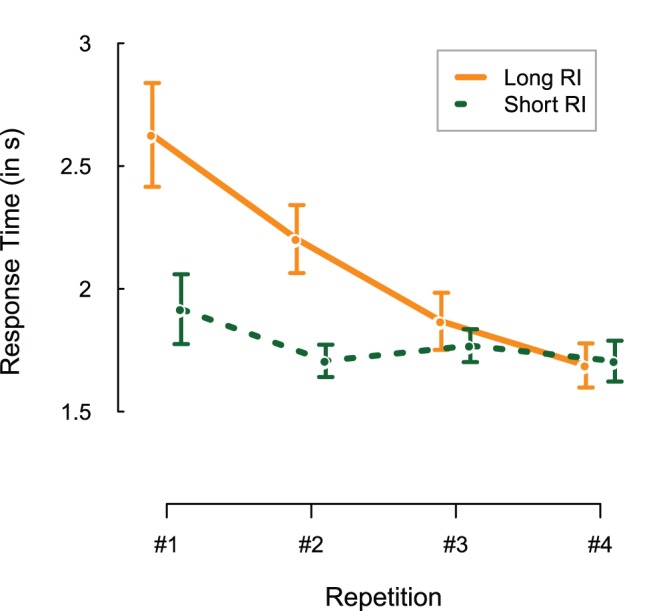
Response latencies per repetition, plotted separately for the long (orange, solid) and short (green, dashed) retention interval (RI) sets. Error bars represent 1 standard error of the mean.

Next, we analyzed the reaction times with respect to the effects of repetition and retention set. Reaction times are measured from the onset of the response screen to the first mouse click. Figure 4 shows the latencies associated with the responses in the long and short retention set conditions. Both main effects and the interaction were significant, as was the intercept (β = 2.32, HPD95 = 2.11, 2.53, p<0.001). The effect of retention set (β = −0.459, HPD95 = −0.62, −0.30, p<0.001) indicates that responses for items in the short retention set were estimated to be 459 ms faster than for those in the long set. The effect of repetition (β = −0.232, HPD95 = −0.28, −0.18, p<0.001) reflects a speedup of about 232 ms per repetition, an effect that is greatly reduced in the short retention sets by the interaction between retention and repetition (β = 0.175, HPD95 = 0.09, 0.26, p<0.001).

The increased performance with increased number of repetitions and the better performance in the short retention sets are in line with the predictions of memory strength theories. Therefore, we take these results as evidence that our operationalization of memory strength was successful.

### Pupillary Data

We will first discuss analyses that are similar to those of correctness and reaction times, with pupil dilation as dependent variable, number of rehearsals and retention set as fixed effects, and participant and item as random effects.

**Figure 5:**
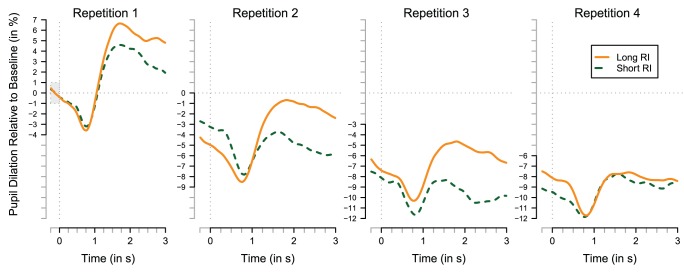
Averaged relative pupil dilation per repetition during the presentation of the retrieval cue, plotted separately for the long (orange, solid; 14 trials per participant) and short (green, dashed; 12 trials per participant) retention interval (RI) sets and aligned to the onset of the presentation of the retrieval cue at t = 0. Pupil dilation is calculated as percentage change relative to the 250 ms before the first repetition, indicated by the grey box in the leftmost panel.

Figure 5 shows the main effects of pupil dilation, expressed as proportion change relative to the baseline. The absolute values for the baseline did not differ for short and long retention sets (average of 968 and 987 arbitrary units [32] for the short and long retention sets; t(14) = 0.95, p = 0.357). The four panels represent the four repetitions of an item, with dashed green lines representing the short retention set and solid orange lines representing the long retention set. A notable feature, present in all four panels, is the initial pupillary constriction immediately following retrieval cue onset. Also, there is a robust decrease (β = −3.24, HPD95 = −4.58, −1.94; p<0.001) in tonic pupil dilation over the repetitions (measured in a window of 200 ms around time 0), which is not influenced by retention condition (main effects and interaction, all p values >.25). This effect is not driven by an overall decrease in pupil dilation during the experiment, because the pupillary response that was measured at the beginning of each of the four experimental blocks (i.e., the baseline as depicted in Figure 5 for the first trial of each of the four blocks) does not decrease (HPD95 = −62.23, 46.27; p = 0.750).

Most relevant for the questions addressed here is whether the task-evoked pupil response fluctuates as a function of retention and repetition. First, there is a main effect of retention set, as the short retention condition is estimated to elicit a 2.6% smaller task-evoked pupil response (β = −2.61; HPD95 = −4.89, −0.33; p = 0.025) when compared to the long retention set (which is captured by the intercept: β = 18.2, HPD95 = 15.4, 20.9; p<0.001). In addition, the task-evoked pupil response decreases about 2% per repetition (β = −1.88, HPD95 = −2.40, −1.40; p<0.001). This decrease is attenuated in the short retention set, as the interaction between short retention set and repetition (β = 1.08; HPD95 = 0.22, 1.87; p = 0.011) reduces the decrease per repetition to −0.80% (i.e., −1.88+1.08) for short retention conditions.

These results are largely in line with the hypotheses: stronger memory traces are associated with smaller evoked pupillary responses.

## Discussion

The goal of the present study was to assess whether and how the memory strength of information retrieved from memory influences pupil dilation while reducing possible confounds associated with response execution. Based on existing literature, we hypothesized that the task−evoked pupillary response would decrease with increasing memory strength, operationalized in terms of the frequency and recency of memory items. The results confirmed the hypothesis, as dilation decreased with increased number of repetitions (frequency) and was smaller for the retention set with a shorter average lag (recency). Since the dilation patterns as shown in Figure 5 clearly indicate that the peak response is long before the onset of the response screen (6 seconds after the onset shown in Figure 5), the retrieval-evoked pupillary response was probably not influenced by any response preparatory processes.

However, a couple of other confounds need to be taken into account. Firstly, the short and long retention conditions are defined by their set size. However, due to the randomization of items at the start of each repetition, the number of intervening items for the long condition might, in extreme cases, be smaller than the number of intervening items for the short condition. Although previous analyses already demonstrated a global effect of recency, a more refined analysis would also include the exact number of intervening trials. Secondly, the onset of the picture with the cross-section of the brain will result in a large pupillary reflex reaction (due to, for example, changes in luminance, color and spatial frequency, see [51]). Since the exposure to the picture depends on the participant’s response latency, reflex patterns might differ per trial. To account for this, we included a factor representing the most recent exposure time to the cross-section of the brain (i.e., the sum of the reaction time and the duration of the feedback of the previous trial starting at trial 2, and an estimated duration of 6 seconds for the presentation of the last study trial for the first test trial).

We started out with a linear mixed-effects model containing, apart from the random effects for participants and items, fixed effects representing main effects and all interactions of the number of repetitions, the two-level factor retention set and the continuous factor number of intervening trials, and a main effect of exposure time. Log-likelihood-based stepwise model selection indicated that including the continuous factor representing the number of intervening trials was not warranted. However, including the exposure time during the previous trial did improve the model fit (χ^2^(2) = 8.16, p = 0.004) but the addition of this additional factor (β = 0.45, HPD95 = 0.15, 0.76; p = 0.004) did not qualitatively affect the estimates of the number of repetitions (β = −1.68, HPD95 = −2.19, −1.17, p<0.001), of the retention set (β = −2.33, HPD95 = −0.10, −4.56, p = 0.044), of the interaction (β = 1.00, HPD95 = 0.17, 1.85; p = 0.017), or of the intercept (β = 15.64, HPD95 = 12.44, 18.87; p<0.001). These results indicate that the exposure time of the cross-section during the previous trial correlates positively with the observed dilation, but that the effects of memory strength as operationalized by number of repetitions and retention have an independent contribution to the pupillary response.

Two other notable features of the pupillary patterns observed in this study (see Figure 5) are the constriction observed after the presentation of the retrieval cue and the decrease in pupil dilation over the four rehearsals. The initial constriction is unlikely to reflect a light reflex, since the change of information on the screen from fixation point to retrieval cue results in a decreased light level. Although many factors have been identified that might result in a pupillary constriction even under conditions of constant luminance (see [42] for a review), a potential cause of this constriction is the accommodation reflex [35]: While there is no direct need to focus on the relatively slow paced presentation of the fixation point, the subsequent presentation of the retrieval cue requires accurate focus. The size of this effect observed here is well within the ranges typically observed for this reflex (e.g., [52]). Another possible explanation is that this contraction is caused by spatial frequency changes [53].

The decrease in average pupillary response over repetitions, easily appreciated in Figure 5 when the pupillary responses at Time 0 for the four repetitions are compared, is not influenced by the experimental manipulation of retention set: for both the small and large retention set, the pupillary response decreases. Given that the retention set affects the retrieval-evoked pupillary response and behavioral measures (accuracy and response latency) as predicted by the memory strength hypothesis, it is unlikely that this decrease over repetitions is driven by a decrease in memory strength. Earlier work that found similar effects has attributed this type of tonic decrease to a decrease in autonomic arousal during an experimental block [54].

To our knowledge, this is the first study that showed task-evoked pupillary effects based on memory retrieval after an online-manipulation of memory strength of individual items, while reducing the possible contaminating influences of response preparation. Yet, the results nicely align with the existing literature that typically focused on memory load (e.g., [3], [4]) or on the pupillary responses to items with a predefined memory strength (e.g., [16], [17]).

Taking into account the relatively slow nature of pupil size changes, participants in our experiment were instructed to retrieve the answer when the retrieval cue was presented, but only to respond after a delay of 6 seconds. This delay allowed us to measure a complete task-evoked pupillary response. The results show that memory strength influences the pupillary reflex before an actual response is given. This suggests that pupil dilation might be used to assess proficiency levels during the learning of factual information without requiring overt learner responses, potentially speeding up learning processes. Moreover, using pupillary deconvolution techniques [37], [38], it should be possible to deduce proficiency levels from pupil dilations in more typical memory paradigms that are characterized by a faster pace. Interestingly, these results corroborate findings by Magliero [55] who observed effects of recency on pupil dilation when varying the time between repetitions of items in a list that had to be committed to memory. When items were presented for a second time, he found that repetitions that followed the initial presentation at shorter lags (i.e., with 0 or 1 intervening trials) resulted in smaller task-evoked pupillary responses than repetitions at longer lags (i.e., with 4 or 8 intervening trials), providing a link between the retrieval processes reported in this study, and the encoding processes studied by Magliero.

To conclude, our findings demonstrate that pupil dilation is an early, online marker of the cognitive effort involved in the retrieval of an item from memory that manifests itself even in the absence of a direct overt response.
